# Microbial Resources as a Tool for Enhancing Sustainability in Winemaking

**DOI:** 10.3390/microorganisms8040507

**Published:** 2020-04-02

**Authors:** Tiziana Nardi

**Affiliations:** CREA—Council for Agricultural Research and Economics, Research Centre for Viticulture and Enology, Viale XXVIII Aprile 26, 31015 Conegliano, Italy; tiziana.nardi@crea.gov.it

**Keywords:** enology, fermentation, *Saccharomyces cerevisiae*, non-*Saccharomyces* yeasts, lactic acid bacteria, bioprotection, wine by-products

## Abstract

In agriculture, the wine sector is one of the industries most affected by the sustainability issue. It is responsible for about 0.3% of annual global greenhouse gas emissions from anthropogenic activities. Sustainability in vitiviniculture was firstly linked to vineyard management, where the use of fertilizers, pesticides and heavy metals is a major concern. More recently, the contribution of winemaking, from grape harvest to bottling, has also been considered. Several cellar processes could be improved for reducing the environmental impact of the whole chain, including microbe-driven transformations. This paper reviews the potential of microorganisms and interactions thereof as a natural, environmentally friendly tool to improve the sustainability aspects of winemaking, all along the production chain. The main phases identified as potentially interesting for exploiting microbial activities to lower inputs are: (i) pre-fermentative stages, (ii) alcoholic fermentation, (iii) stage between alcoholic and malolactic fermentation, (iv) malolactic fermentation, (v) stabilization and spoilage risk management, and (vi) by-products and wastewater treatment. The presence of proper yeast or bacterial strains, the management and timing of inoculation of starter cultures, and some appropriate technological modifications that favor selected microbial activities can lead to several positive effects, including (among other) energy savings, reduction of chemical additives such as sulfites, and reuse of certain residues.

## 1. Introduction

In agriculture, the wine sector is one of the industries most affected by the environmental sustainability issue. On a global scale, it is responsible for around 0.3% of annual global greenhouse gas (GHG) emissions from anthropogenic activities; this corresponds to about 2% of agriculture contribution, which in turn is estimated to contribute to 14% of the total anthropogenic-derived GHG emissions [1,2]. 

The mounting interest regarding the environmental impact of wine production and its potential to modify regional climate patterns has prompted many wine producers to move toward sustainable grape growing and wine production practices. Moreover, recent analyses of consumer perceptions, preferences, and willingness to pay for wine showed that producing and marketing wine with sustainability features is a promising strategy for quality differentiation, providing an additional stimulus for the wine industry to proceed toward a larger adoption of sustainable practices [3]. Therefore, improvements in terms of energy and water consumption, use of pesticides and additives, and the polluting effects that these inputs may have on the biosphere, together with their potential concern for consumer acceptance, have prompted recent research activities.

The exploitation of microbial resources to improve sustainability of the winemaking process, nonetheless, is a very recent approach and only a few research studies have addressed it. Sustainability in vitiviniculture was first linked to vineyard management, where crucial issues are related to environmental emissions arising from the use of fertilizers, pesticides, and heavy metals [4,5]. Afterward, some research surveys performed under EU programs and via industry audits [1,4,5] assessed the environmental impact of each input used all along the wine production chain, including winemaking phases. Consequently, several programs for wine life cycle assessment (including initiatives following the European Regulation EMAS [6]) accounted, among other factors, equivalent emissions for electricity consumption and for sulfur dioxide used in the vinification phase [7], which are in turn influenced by microbial transformations and their management.

In this regard, one of the aspects of a two-way relationship between microorganisms and climate change in winemaking has been recently addressed and reviewed: the microbial potential as an adaptation strategy for the effects of climate change [8,9,10]. The abovementioned papers described the potential of pro-technological microbes as agents capable of mitigating the negative features of the evolving climatic influence (e.g., microbial solutions to reduce ethanol content, improve organic acids content, and reduce pH). However, the microbial potential for reducing the environmental impact of winemaking (and therefore for lowering the contribution of wine production to climate change) has not been comprehensively described to date. The aim of this paper is to review the potential of microorganisms and interactions thereof as a natural, environmentally friendly tool to improve the sustainability aspects of winemaking, all along the production chain, in different phases. Microorganisms play a role in several steps of the winemaking process, from grape harvest to bottling and waste treatment, and most of these steps can be improved for reducing the environmental impact of the whole chain, including fermentation stages. In this context, the exploitation of microbial resources and the best management of their interactions will be considered for their potential effects on the environmental impact of winemaking, including (i) limiting the use of chemical additives such as sulfites used as preservatives against microbial spoilage, taking advantage of beneficial microorganisms’ activities; (ii) allowing a better energy management and achieving energy savings during fermentations; (iii) avoiding additional inputs such as filtrations, cellulose-based adjuvants, additional warming and cooling steps, and other technological corrections that are eventually used in case of stuck or sluggish fermentations; (iv) improving, in certain cases, microbial biodiversity; and (v) valorizing and/or reducing the environmental impact of wine industry by-products and wastewater. Special attention will be paid to microbial resources and processes which are already, or about to become, available for the winemaking sector at the industrial scale.

## 2. Improving Sustainability in Pre-Fermentative Stages 

**Figure 1:**
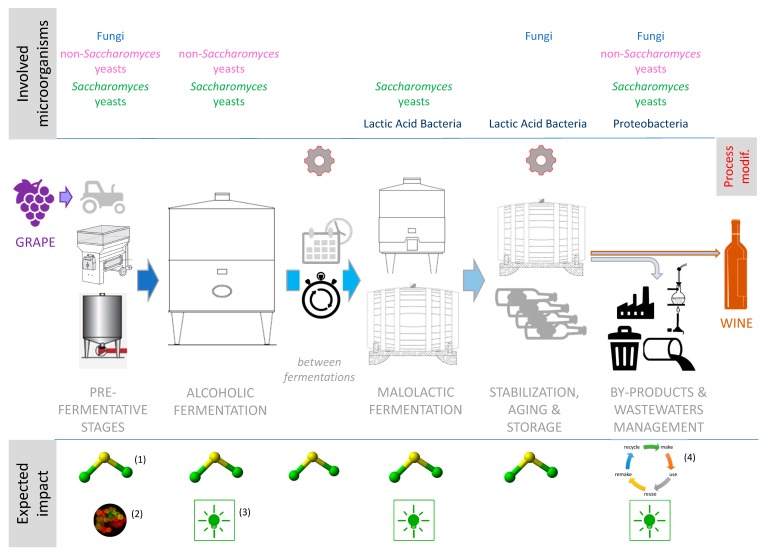
Scheme showing the different winemaking phases and displaying the critical points considered in the paper for enhancing sustainability. Symbols legend: (1) Sulfite reduction, (2) biodiversity improvement, (3) energy savings, (4) reuse and valorization of by-products.

Grape transport after harvest and grape crushing are typically the first steps where juice is released and therefore, sulfite is usually added for its antioxidant, antioxidasic, and antimicrobial properties [11]. The antimicrobial effects reduce the activity of “apiculated” yeasts and some bacteria that are present on the grapes and can develop in the cellar, causing off-flavors and defects either before or after fermentations [11,12,13,14]. In this context, the early inoculation of grapes or must with different yeasts has developed considerably in recent years in order to bioprotect the must by directly colonizing the environment and preventing the development of spoilage microorganisms. The industrial objective is to reduce the dose of sulfites and to substitute their effect as much as possible [15,16,17,18], as displayed in Figure 1. Early yeast inoculation, which is also recommended by the International Organisation of Vine and Wine (OIV) as an useful practice to achieve this goal [19], was first intended for moving up the timing of *Saccharomyces* addition for launching alcoholic fermentation (AF) [20]. More recently, the early inoculation of yeasts belonging to the heterogeneous group of non-*Saccharomyces* genera is also becoming a common and innovative practice. Indeed, not only the role of non-*Saccharomyces* yeasts in winemaking has been re-evaluated for their benefits on the quality and sensory properties of wine (reviewed in [21,22,23,24,25]), but also for the additional advantage they provide for must bioprotection by directly colonizing the environment. Even though these yeasts do not necessarily play a role in sugar fermentation, they contribute to preventing the development of undesired microorganisms [15,16,18]. It is worth to note that limiting sulfite addition at this stage can, at times, generate a double advantage: it can indirectly have a positive effect on the production of SO_2_ by *Saccharomyces* yeasts that will later on ferment the sugars. It is known that certain enological strains can overproduce this molecule in the presence of high starting concentrations in the must [26].

This approach can, in certain cases, also increase microbial biodiversity. The use of a wide range of yeasts (non-*Saccharomyces* and *Saccharomyces*) and lactic acid bacteria (LAB) as starters can tackle the problem of uniformity caused by the use of a few active dry yeast (ADY) strains when this practice was first introduced (in the 1960s) [13]. Some winemakers have tried to increase the influence of the native yeasts by delaying or reducing the use of starter cultures. However, this can lead to uncontrolled fermentations [14]. The noncontrolled fermentations might lead to economic losses due to the risk of spoilage and are hardly compatible with sustainable sulfite management for wine stabilization. The alternative proposed by a recent approach is to incorporate the features of “native” microorganisms to starter cultures: the use of a wide array of different cultures aims at reproducing the vineyard natural microbiota, exploiting different strains as well as different species [14,15]. As the range of available microorganisms increases over time, this gives winemakers the possibility to increase biodiversity also in guided fermentations [16], as reported in Figure 1. The selection of local or autochthonous strains to be included in the starter cultures would further enhance this possibility [17], meanwhile aiming at bioprotecting the must [27].

Focusing on non-*Saccharomyces* yeasts, the goal of grape and must microbial bioprotection can be achieved through the exploitation of different properties thereof, including (i) specific bioprotective features conferred to certain species and strains by their ability to produce molecules with antimicrobic activities, such as killer toxins or pigments with antifungal action and (ii) more generally, an effective competition for nutrients with natural microbiota that prevents the development of other microorganisms, including possible spoilers. Moreover, a side effect that also contributes to sulfite substitution is potential protection against oxidation; this could be exerted by selected non-*Saccharomyces* yeasts due to their rapid consumption of oxygen, which prevents oxygen utilization by oxidative yeasts [16].

### 2.1. Non-Saccharomyces Yeasts Producing Antimicrobial Compounds

One of the most interesting, studied and discussed yeasts to be inoculated in early stages of winemaking (on grapes or on must in pre-fermentative stages or at the beginning of alcoholic fermentation, a few hours before a *Saccharomyces* strain) is the genus *Metschnikowia*, with its species *Metschnikowia pulcherrima* and *Metschnikowia fructicola*.

*Metschnikowia pulcherrima* can be used as a biological control agent due to its ability to produce natural antimicrobial compounds, namely pulcherrimin, an insoluble red pigment with antifungal activity. This peculiar antimicrobial activity is produced by the depletion of iron in the medium through the precipitation of iron(III) ions caused by interaction with pulcherriminic acid, a precursor of pulcherrimin secreted by *M. pulcherrima* [28]. In this way, the environment becomes inhospitable to other microorganisms that require iron for their development. Pulcherrimin has shown effective inhibitory activity against several yeasts: *Candida tropicalis*, *Candida glabrata*, and *Candida albicans*, as well as yeasts belonging to *Dekkera/Brettanomyces*, *Hanseniaspora*, and *Pichia* genera [28,29,30]; and fungi: *Botrytis cinerea*, as well as *Penicillium, Alternaria, Fusarium, Rhizopus*, *Verticillium*, and *Monilia* spp. [30]. *Metschnikowia pulcherrima* has therefore been described as a biofungicide capable of effectively reducing the incidence of *Botrytis* development in post-harvest fruits, such as apples [31,32,33], citrus [34], and cherry [35]. In most cases, its antagonistic mechanism is completed by its competition for nutrients [28]. Some research papers have proposed the application of *Metschnikowia pulcherrima* on grape berries [36,37,38], since *S. cerevisiae* seems not to be affected by *M. pulcherrima* antimicrobial activity [29], so the use of this yeast as a selected starter at any stage prior to *Saccharomyces* inoculation is compatible with alcoholic fermentation [28]. To our knowledge, the most industrially relevant enological applications of this species are proposed by some companies for protecting must and controlling the indigenous microbiota (pre-fermentative control and bioprotection), either alone or in association with *Torulaspora delbrueckii* [39], with early-stage inoculation (grape bunches before crushing and grape must after crushing). Moreover, its application in pre-fermentative stages (namely, pre-fermentative cold maceration, PCM) has been tested in sulfite-free must at the winery level and also compared with strains of *Metschnikowia fructicola* [40,41]. Although these studies were set up for elucidating the positive effects of *Metschnikowia* on chromatic and aromatic wine characteristics, the results also indirectly confirmed the potential of this species for bioprotection purposes in early winemaking stages, by showing good implantation and population dynamics in PCM.

Besides *Metschnikowia*, other yeast species have a broad killer spectrum against spoilage yeasts, including, among others, *Wickerhamomyces anomalus* (formerly *Pichia anomala*), *Kluyeromyces wickerhamii*, and *Torulaspora delbrueckii* [42,43,44]. After its initial discovery in *S. cerevisiae*, the killer phenotype was described in non-*Saccharomyces* yeasts [45] and consequently, later on, killer toxins have been proposed as a biocontrol strategy alternative to the use of chemical preservatives or physical methodologies during the winemaking process [43,46]. Killer toxins are generally defined as antimicrobial proteinaceous compounds that inhibit susceptible yeast species or strains, although they remain immune to their own toxins [46]. Although killer proteins have been reported as antimicrobial agents against diverse undesired microorganisms present in different foods [43], they are mainly tested against the prevailing wine spoilage microorganisms *Dekkera/Brettanomyces* in the enological environment [17,46]. Despite this wide diversity, the killing action of all of the characterized killer toxins is generally mediated by a two-step mechanism, where cell wall is generally the primary site of action. Therefore, cell wall components such as β-1,3-D-glucans and β-1,6-D-glucans are common receptors for the majority of killer toxins characterized to date, although mannoproteins and chitin also serve as first receptors for a number of killer toxins [43]. Recent studies have focused on Kwkt and Pikt, zymocins produced by *Kluyeromyces wickerhamii* and *Wickerhamomyces anomalus*, respectively, with antimicrobial properties [47], and on TdKT produced by *T. delbrueckii* [48]. Recently, the killer toxin KTCf20 secreted by the strain *W. anomalus* Cf20 was also suggested to bind to β-1,3 and β-1,6 glucans of the cell wall of sensitive strains [42]. Interestingly, these killer toxins are not affected by the pH, temperature, and ethanol concentrations that are typical of winemaking conditions. Furthermore, they do not inhibit the fermenting *S. cerevisiae* strains or the lactic acid bacteria and are therefore hypothesized not to have a negative impact on alcoholic and malolactic fermentation [43]. Thus, it was hypothesized that the use of *W. anomalus* starter cultures can partially replace SO_2_ during grape must fermentation, in order to reduce the wine sulfite content; antimicrobial activity was also reported toward other minor yeast species present during the early stages of grape fermentation, such as *Pichia guilliermondii* or *Pichia membranifaciens* [17,42]. Moreover the potential use of the purified toxin Pikt from *W. anomalus* D2 as an alternative to sulfur dioxide (SO_2_) has been proposed because Pikt, unlike SO_2_, produced irreversible damage on sensitive yeasts, ensuring the complete control of spoilage *Brettanomyces* yeasts [42,47,49].

Nevertheless, it is worth to note that none of these microbe-based innovations (neither killer yeast starter cultures nor purified toxins thereof) are currently used in winemaking at the industrial scale. Indeed, to our knowledge, there are no suppliers in the enology sector proposing these solutions anymore, although for a while, a company used to sell *W. anomalus* and *K. wickerhamii* starter cultures in fresh cream [39,50]. Several reasons may explain this situation, including cost-effectiveness issues. Beyond their undoubtful antimicrobial activity, protective cultures/toxins should be produced at moderate costs in order to make them available (in adequate concentrations for industrial winemaking) at affordable prices for wineries.

### 2.2. Non-Saccharomyces Yeasts Exerting Indirect Bioprotective Effect

Besides specific antimicrobial activities, biocontrol strategies are based on the activity of living microorganisms that counteract the evolution and harmful effects of spoilage microorganisms, without interfering with the life cycle of useful microorganisms or creating risks for human health. Concerning grape bunches and berries, some biocontrol agents, including *Aureobasidium pullulans*, *Metschnikowia pulcherrima*, and *Pichia guilliermondii*, have been proposed against *Botrytis cinerea* to protect fresh fruit and table or wine grapes [32,37,38,51]. Moreover, some authors examined the microbiota associated with dried grapes in traditional wine production as a source of biocontrol agents against *B. cinerea* and found interesting activities in several other genera, including *Hanseniaspora*, *Cryptococcus*, and *Issatchenkia* [52], together with epiphytic bacteria mainly belonging to the *Bacillus* taxon [53]. In another recent work, fermenting must obtained from overripe grape berries and therefore more susceptible to fungal infection was considered for the selection of yeasts with antifungal activity. Promising antifungal activity against *B. cinerea* was demonstrated in *Starmerella bacillaris* species and the production of volatile organic compounds (VOCs), tested in vitro, was found to be mainly responsible for the observed effects [54].

To our knowledge, the most industrially relevant enological non-*Saccharomyces* yeast used in this context is a strain of *Metschnikowia fructicola*, a species also known for its post-harvest biocontrol potential on other fruits [55,56]. In particular, the abovementioned strain is currently being considered for withering of grapes [57], in order to biocontrol *Botrytis* infection during the natural drying process of grape bunches for “passito” wine production. Since *S. cerevisiae* seems not to be affected by *M. fructicola* presence, the use of the latter as a selected starter in all the pre-fermentative stages, including cold macerations, has gained great interest in modern enology, both for the many benefits on wine quality associated with this yeast and for the possibility of working with low sulfite doses [40,41].

During the crushing stage, the strategy to bioprotect must as soon as possible at the biochemical level, avoiding undesired microorganisms’ metabolism, by inoculating selected starter cultures is increasingly being employed [18]. Indeed, it has been shown that some non-*Saccharomyces* species support or inhibit the growth of other non-*Saccharomyces* and *Saccharomyces* species in multispecies consortiums, and that the relative performance of each yeast species is dependent on its fermentation capacity, initial cell density, and ecological interactions as well as tolerance to environmental factors [58]. A large number of research work has addressed this topic in recent years, most of which focused on the limitation of spoilage occurrence, mainly due to *Brettanomyces bruxellensis* growth (for reviews, see [17,39,59]). Narrowing the field to those studies that tested strains and strategies at the winemaking scale (pilot or industrial), interesting results about yeast inoculation were observed from a trial aimed at understanding the impact of a *Torulaspora delbrueckii* strain used as a bioprotective agent instead of sulfite addition. The authors demonstrated the effects of the *T. delbrueckii* strain, inoculated at the beginning of the white winemaking process, in two Burgundian wineries and proposed it as an alternative to the use of sulfites [16]. A further improvement of this strategy is the use of both non-*Saccharomyces* yeasts and lactic acid bacteria for obtaining must and minimizing the sulfur dose [60,61], as recently shown in a study that reported the sequential inoculation of *Lactobacillus plantarum* and *Lachancea thermotolerans* as a promising winemaking alternative in contrast to traditional vinification and also showed the advantage of producing wines with higher titratable acidity and lower pH [18].

The indirect antagonistic activity of selected non-*Saccharomyces* yeasts on undesired wine yeast species is considered to be a key property for many starter cultures that display possible biocontrol applications, but are also interesting for the winemaker as tools, for improving the sensory properties of the wine, that are susceptible to modulate the sensory profile/volatile aroma composition and/or exploitable to modify acidity or color in wine [8,39,41,60]. Therefore, the potential application of starter cultures of species which harbor bioprotective potential (either direct or indirect) coupled with other enological features seems to be wider than the application of species that do not bring any technological/sensory property other than bioprotection through specific antimicrobial mechanisms (e.g., killer toxins).

## 3. Managing Alcoholic Fermentation for Sustainability

The use of selected starter cultures to “guide” grape must fermentations was first introduced by inoculating *S. cerevisiae* yeasts with the aim of securing a rapid and reliable fermentation process, avoiding organoleptic defects and obtaining wines with desired quality and sensory characteristics [20]. The different potentialities of this step for improving sustainability are described below and summarized in Figure 1.

### 3.1. Control of Microbial Spoilage during Alcoholic Fermentation

Concerning the impact of alcoholic fermentation (AF) management on sustainability, that is, on the reduction of sulfites and other interventions linked to the risk of microbial spoilage, important guidelines have been provided by the OIV, in 2014, within the resolution “Code of good vitivinicultural practices in order to avoid or limit contamination by *Brettanomyces*” [62]. The document explains that *Brettanomyces* can grow as AF slows down or stops and therefore, enological practices commonly recommended for the management of alcoholic fermentation must be implemented, including inoculation of must with selected yeasts that help to achieve a more reliable AF. The fact that the environment becomes more favorable to the multiplication of *Brettanomyces* if alcoholic fermentation slows down or stops is pointed out, and in the case of the latter, using a process to restart alcoholic fermentation as soon as possible is recommended (as endorsed also in reference scientific studies) [63]. Finally, the OIV document recalls that residual sugars (mainly glucose and fructose) are substrates for *Brettanomyces* growth [62] and therefore, their leftover should be avoided by carefully monitoring the completion of AF. All the above mentioned goals can be achieved through an effective management of AF, which is usually pursued by taking advantage of a fruitful inoculation of *Saccharomyces* starters [61,64] and with careful management of nutrient supplementation, including both nitrogen and lipid-based molecules [65,66].

Besides the inoculation of a robust strain able to consume sugars, performed early enough to avoid the development of undesired indigenous microorganisms, the choice of a *Saccharomyces* starter strain can bring along some other features useful for reducing the final amount of sulfites in wine. The proper choice of wine yeasts and bacteria is a key factor in determining the final levels of both SO_2_ and acetaldehyde produced [26,67,68]. Acetaldehyde is a key component of wine, formed by yeasts during alcoholic fermentation, that can bind with SO_2_, and since wines with high levels of acetaldehyde require more exogenous SO_2_, this can be a concern [69]. At the same time, *Saccharomyces* yeasts, including commercial starters, widely differ for SO_2_ production: the production of sulfites by wine yeasts is highly strain-dependent, and despite strong selective processes, some commercial yeast still produce high amounts of these sulfur compounds in some circumstances [67]. Therefore, a correct and careful choice of the *Saccharomyces* strain for alcoholic fermentation is a key issue for managing acetaldehyde and SO_2_ production during the winemaking process [70,71,72]. Wine yeasts selected or breaded for having low SO_2_ and acetaldehyde production are, in this context, a valuable tool for achieving the abovementioned goal [73]. Besides *Saccharomyces* wine yeasts, selected strains of non-*Saccharomyces* species can represent a further improvement: in a recent work, *Starmerella bacillaris*, used in sequential inoculation with *S. cerevisiae*, produced less SO_2_ and acetaldehyde compared with *Lachancea thermotolerans* and *Metschnikowia* spp. or with *S. cerevisiae* alone [74].

Moreover, the production of sulfites depends on environmental factors, including the concentration of nutrients in the media, in particular, nitrogen-containing compounds (ammonium, amino acids and especially sulfur-containing amino acids) [75], and on starting sulfite levels [26,75]; therefore, the management of alcoholic fermentation (including the choice of yeast nutrients) might be a tool for modulating SO_2_ production by yeasts.

Additionally, the careful choice of yeast strain and nutrition protocol will also bring benefits for winemaking stages, mainly malolactic fermentation. Low-SO_2_-producing strains usually do not inhibit malolactic fermentation (MLF) and therefore, favorize further stages (see par. 5).

### 3.2. Energy Savings Associated with Alcoholic Fermentation

Literature has extensively described the effect of temperature on yeast metabolism during wine fermentation [76]. As shown in the last decade [76,77,78], the effect of low temperature on fermentation efficiency and aroma production varies markedly for different *S. cerevisiae* strains.

Certainly, temperature control during fermentation significantly impacts the energy demand of wineries. The majority of the electricity used by wineries (about 90%) is consumed by refrigeration systems for process cooling, that is, fermentation control, cold stabilization, and cold storage [79,80]. The fermentation process takes place at a controlled temperature for quality purposes, to which the wine needs to be cooled at the beginning of fermentation and throughout the process; and the fermentation reaction also generates heat that needs to be removed [79]. As expressed before, the increased interest of consumers in the environmental aspects of winemaking, combined with economic pressure, compels winemakers to address concerns over energy consumption during wine production and to identify potential energy savings. Moreover, the gained awareness among retailers and distribution chains will drive wine suppliers to provide quantitative information on their energy saving solutions and their impact on the environment thereof [81,82,83]. As a consequence, recent research studies addressed the quantification of required heat dissipation during AF, showing interesting results [84,85].

In an initial work, a newly selected *Saccharomyces* wine strain was tested in the production of sparkling base wine, fermented at a temperature higher than the winery standard. The quantification of electric energy consumption and estimation of energy conservation showed that increasing the temperature from 15 °C to 19 °C during the fermentation process yielded an energy saving of ~65% [84]. No significant differences were found in the main chemical wine parameters and sensory characteristics (through a triangular panel test) between the two temperatures. This was consistent with volatile compound quantitation, as only 25% of the tested aromatic molecules showed a change in concentration with the fermentation temperature (most of them were higher at 19 °C than at 15 °C), and many were below the sensory threshold. Moreover, no measurable SO_2_ was produced by the yeast (low-producer strain) in any of the fermentations, confirming that temperature did not affect sulfite production for this specific strain at the tested conditions. This study was the first to quantify energy conservation from sustainable temperature management during base wine fermentation, showing the benefits of such an approach [84].

In a more recent work, required heat dissipation was measured in Riesling fermentation and the results confirmed and further illustrated the relevance of the temperature program employed with regard to energy demand for cooling [85]. Approximately 70% less heat had to be dissipated for fermentation at 19 °C, compared with that for fermentation at 14 °C. Approximately 30% less heat had to be dissipated under a 16–11–17 °C temperature program, compared with that for fermentation at 14 °C. Thus, high savings in electrical energy can be expected, although depending on the technical configuration of the cooling system. The formation of most esters was more pronounced in the second half of fermentation at higher temperature. No difference was found in the final concentration of acetate esters or acetic acid. Acetaldehyde concentration was 35% lower for fermentations at 19 °C, compared with those at 14°C. A descriptive analysis, at 5 and 11 months after bottling, revealed no significant difference in wine sensory profiles.

Overall, these studies, carried out with different selected yeast strains, show that energy savings can be achieved by reducing the required dissipated heat through temperature management of fermentations, without compromising wine composition. Yeast characteristics and expected aromatic profile should be carefully considered as the strain choice criteria, when deciding on temperature management and related saving potential. Moreover, in the reported studies, the use of innovative thermal protocols allowed the wineries to adopt more sustainable winemaking processes with low SO_2_ [84] and acetaldehyde [85] production together with low energy consumption, and consequently, to propose ecolabeling strategies and price premium policies that presently have marketing benefits [3].

## 4. Improving Sustainability between Alcoholic and Malolactic Fermentation

In modern winemaking, the timing of occurrence of malolactic fermentation (MLF, traditionally happens during storage) is advancing, taking place right after AF or even during AF when co-inoculated is used. This is due to climate change, which determines modifications in grape/must composition such as pH increase, as well as an enlarged need to better manage this step and avoid risky situations in the time frame between AF and MLF [8,61]. Indeed, wines waiting for MLF cannot be stabilized and, in some situations, need to be warmed to favorize bacterial development, but these situations also favor the growth of microbial spoilers. As such, early MLF management has been recommended by the OIV as a good winemaking practice to avoid wine spoilage that causes major economic losses [19] (outlined in Figure 1).

Indeed, MLF management strongly affects the development of spoilers, mainly *Brettanomyces*, during subsequent wine aging. Some studies showed that wines that underwent rapid MLF inhibited the growth of *Brettanomyces*, resulting in a product containing little or no volatile phenols. Conversely, wines that did not undergo MLF or underwent late spontaneous MLF that proceeded slowly allowed the proliferation of *Brettanomyces*, resulting in a product containing more volatile phenols [86,87]. The abovementioned OIV resolution [19] attests that if MLF is delayed, the risk of production of volatile phenols increases because *Brettanomyces* can take advantage of the time between alcoholic and malolactic fermentations to multiply, benefiting from the absence of SO_2_. Thus, the use of malolactic starters is proposed as a good way to limit *Brettanomyces* development and volatile phenol production. Co-inoculation or early sequential inoculation is presented as the best tool to prevent *Brettanomyces* contamination by reducing the lag phase between AF and MLF, as also shown in scientific studies [86,88].

## 5. Managing Malolactic Fermentation for Sustainability

MLF is the microbial transformation that, more than others, affects post-fermentation stages such as aging, color stabilization, and microbial stabilization. As expressed before and also outlined in Figure 1, an effective and reliable MLF with no lag phases and no nutrient leftovers is an essential step for avoiding further microbial spoilage and consequent overutilization of sulfites or other corrective tools [89,90]. Moreover, stuck or sluggish MLFs not only put the final wine quality at risk, but also require enological interventions, such as additional racking-off operations, cellulose-based adjuvants, and cooling or warming (depending on the situation), that burden the environmental impact of the winemaking process. In this context, the current knowledge on yeast–bacteria characteristics and interactions can be implemented by winemakers in protocols for avoiding fermentation slowdowns in risky situations.

### 5.1. Control of Microbial Spoilage during Malolactic Fermentation

Over the last 20 years, various studies have reported many factors that influence the development of LAB in wine, providing to winemakers an interpretation key to understand MLF problems and some tools to manage the MLF process [89,91,92,93,94]. Indeed, if some of the parameters affecting MLF feasibility are not easy to change (grape variety, alcohol or potential alcohol, pH, and malic acid content), many others can be managed by the winemakers in order to minimize risks of stuck or sluggish fermentations (SO_2_ concentration, temperature, nutrients, and yeast strain), as reviewed in [61]. Moreover, difficulties arising from the impact of two or more of the abovementioned conditions together may cause a problem of much greater difficulty than what would have been predicted by a single parameter acting alone. Therefore, each step of the winemaking process needs to be approached with as complete an understanding as possible to favor bacterial development and fermentative activity. Among other factors, the yeast strain chosen for AF deserves a special attention in this paper, as yeast–bacteria compatibility is a key parameter for exploiting microbial resources and their interactions for a fruitful MLF [95,96]. The ability of LAB to undergo MLF is affected by many factors directly or indirectly influenced by the yeast strain carrying out AF, including inhibitor content (e.g., SO_2_ and medium chain fatty acids), nutrient consumption/limitation, other potential as-yet-unknown factors, and interactions with the indigenous microflora of the fermentation [97]. Therefore, the use of yeast strains known to inhibit MLF should be avoided, despite their potential enological interest (e.g., for aromatic or fermentative features) at least in difficult must conditions [98]. Various studies have addressed the interactions between bacteria using different yeast/bacteria pairs, as summarized in earlier reviews [61,91,95,96], giving the winemaker useful information for strain choice. Particular attention to yeast strain individuation should be paid when using non-*Saccharomyces* and *Saccharomyces*, in order to keep the environment favorable to MLF later on [60,99].

For successful induction of malolactic fermentations when starter cultures containing malolactic bacteria are used, it is critical that the most appropriate bacterial strain is selected for the prevailing wine conditions. Since the four main limiting factors (alcohol, pH, temperature, and SO_2_) have a cumulative stress effect on cultures, all should be considered for the best choice [61,100]. This may lead to the choice of inoculating either an *Oenococcus oeni* or *Lactobacillus plantarum* strain, depending on the must condition. Another point on MLF management that can be important to prevent spoilage is biogenic amine (BA) formation. Previous work showed that most of the commercial malolactic bacteria did not produce BA, and that the application of commercial malolactic starters in wines is useful to reduce the BA amounts, since BA concentrations in inoculated wines were significantly lower compared with those in non-inoculated wines [101]. These results suggest that the use of selected malolactic starters can minimize BA production [102]. When BA-producing strains are present in indigenous microbiota, a winemaker is particularly encouraged to inoculate selected malolactic starters to replace the indigenous microorganisms. Nevertheless, when the dominance of starter cultures on the indigenous BA-producing microbiota is not sufficient, this does not represent the definitive solution. Thus, a recent study reports the selection of autochthonous strains of *L. plantarum* able to degrade BA and their suitability as malolactic starters in wine production [103]. This represents one more scenario in which BA could be controlled, in fermented foods, by modulating microbial resources as MLF.

### 5.2. Energy Savings Associated with Malolactic Fermentation

As inoculated fermentations of both yeast and bacteria are nowadays practiced in most wine regions of the world, there has been considerable research aimed at optimizing the time point for the inoculation of different yeasts for AF and bacteria for MLF, the latter resulting in a growing interest in the use of co-inoculation (inoculation of LAB starters at 24 to 48 h after yeast inoculation) in the production of many red and some white wines [104]. As expressed before (Section 4), co-inoculation is often proposed as a worthwhile alternative for winemaking compared with traditional post-alcoholic fermentation LAB inoculation or spontaneous MLF [105] (as more and more findings illustrate that co-inoculated MLF is an effective and novel way of modulating the volatile and aroma compound profile of wine [104,106,107]). Indeed, relative to sequential inoculation, co-inoculation reduces overall vinification time. This has at least two important consequences for the wine industry; firstly, speeding up the vinification rate leads to more rapid wine stabilization and reduces the risk of spoilage [90,104], as also recommended by the OIV [19]; secondly, this can significantly reduce the necessity to heat tanks or the whole cellar, a step that is necessary to start the MLF when a sequential inoculation or spontaneous MLF is desired. The heat that is naturally produced by yeasts during alcoholic fermentation favors malolactic fermentation (MLF), thus the early management and accomplishment of malolactic fermentation allows to avoid tank warming, necessary to achieve malic acid transformation in winter/spring season [61]. In this context, more research will be needed to accurately quantify energy savings related to co-inoculation, as less data are currently available. Some rough estimations have been published in Italy in 2009 [108], in a study where total money savings from co-inoculation (including mainly the cost for energy saved for warming tanks, but also extra costs kept back, such as wine analyses) were estimated as €0.08/bottle. In the frame of an ongoing European research project, a study was carried out in Spain comparing controlled malolactic fermentations using co-inoculation with spontaneous MLF [109]. The wines were kept at 20 °C until MLF was completed. Preliminary results showed that the co-inoculated MLF was very fast (completed 5 days after the end of AF), whereas the spontaneous tank started MLF very late (completed 45 days after the end of AF). In this long period of time, the energy consumption to heat the tank was measured and resulted in a significant value, in the order of 150 kWh/hL. This energy expenditure for the spontaneous MLF trial had a calculated cost in the order of €10/hL, although cost may vary according to the price of kWh depending on the country, the power of the heating equipment, the outside temperature, the volume of wine, and the duration of MLF [109,110].

## 6. Sustainable Procedures in Post-Fermentation, Stabilization, Aging, and Storage

As stated before, sulfur dioxide (SO_2_) is the key additive for the preservation of wines; therefore, its role is not limited to must treatment, but is also crucial for stabilizing wines in post-fermentative stages [11]. In this context, the correct management of fermentation steps (alcoholic and malolactic) can be critical to obtain the best ratio between free and bound SO_2_. Beyond the contribution of AF (production and consumption of both SO_2_ and its binding-molecule acetaldehyde by yeasts, described in Section 3.1), late stages of malolactic fermentation have been known to have an impact on bound SO_2_, potentially reducing its levels. Some studies suggest that microbiological wine stabilization at one week after malic acid depletion is an effective strategy for maximum removal of SO_2_ binders. This time frame is optimal for exploiting acetaldehyde consumption by *O. oeni*, still controlling the risk of possible post-MLF spoilage by bacteria leading to the production acetic acid and biogenic amines [111,112].

In case of microbial detrimental contamination, the addition of chitosan is another microbe-based tool for controlling the growth of undesirable microorganisms, particularly *Brettanomyces* [113], but also acetic acid bacteria [114]. Chitosan is a linear polysaccharide composed of two repeating units (D-glucosamine units (GlcN) and N-acetyl-D-glucosamine (GLcNAc) units) randomly distributed along the polymer chain and linked by β(1-4)-bonds [115]. The chitosan preparation specifically allowed in winemaking is microbial (“of fungoid origin” [116]) and is currently produced in *Aspergillus niger* [113]. Recent studies have shown the impact of chitosan application on wines contaminated with *Brettanomyces bruxellensis*, leading to a drop in *B. bruxellensis* cells, even at population levels as high as 10^5^–10^6^ CFU/mL [117]. In some studies, the chitosan preparation was added to the wine under storage and the wine was racked off (usually after 10 days), and the efficiency of the treatment was evaluated in a short delay after the wines were racked off [118,119]. In other cases, chitosan was successfully employed to control wine microbiological stability during the period of aging in barrels, in order to prevent wine from *B. bruxellensis* contamination along the aging period (up to 9 months) [87].

Furthermore, some investigations may propose in the future new strategies to select *Oenococcus oeni* strains holding competitive advantages for surviving in wine after fermentation, preventing microbial spoilage, and improving the wine organoleptic profile, thanks to their biofilm formation. Indeed, prior observations showed that *O. oeni* was able to survive for several months in harsh wine conditions in oak barrels. Since biofilm is a prevailing microbial lifestyle in natural environments, the capacity of *O. oeni* to form biofilms was recently investigated on winemaking materials, such as stainless steel and oak chips [120]. Promising results showed that biofilm could be considered as a novel approach for performing MLF and as an alternative way of adapting MLF starters to wine stress.

## 7. Sustainable Management of By-Products and Wastewater

Bioeconomy and circular economy have gained political traction during the second decade of this century. The movement of bioeconomy toward the use of wastes, co-products, and residue sources resonates well with circular economy principles of making the most efficient uses of natural resources. Microbial transformations, such as fermentations for metabolite production, composting, and controlled oxidizations may contribute to this effort in the case of winemaking for transforming co-products and by-products, including grape marc and pomace, vine shoots, and winery wastewater (as outlined in Figure 1). Indeed, up to 210 million tons of grapes (*Vitis vinifera* L.) are produced annually, with 15% of the produced grapes addressed to the wine-making industry. This socio-economic activity generates a large amount of wastes (up to 30%, *w*/*w* of the material used) [121].

### 7.1. Microbial Valorization of Solid Co-Products

Grape marc is the most important by-product of the winemaking industry. It consists of the solid residue left after juice extraction from grapes and contains skins, seeds, and, in some cases, stalks. It can represent a co-product to be further valorized or a waste to be treated, depending on the situation. Spirits obtained from grape pomace distillation are produced in almost all the Mediterranean countries, allowing economical valorization of marc, which can therefore represent a co-product in this area. The fermented material to be distilled is usually produced by extended storage of the marc, which allows alcoholic fermentation, therefore involving a further microbial transformation, especially in the case of white pomace [122]. Grape marc from red grapes has already undergone alcoholic fermentation with the must and can be distilled immediately, whereas marc from white grapes does not contain ethanol, but contains sugars that are fermented by spontaneous anaerobic fermentation during a storage period. Marc is stored for a period lasting from a few days to several weeks, when fermentation of residual sugars occurs mainly by yeast activity, but bacterial populations can also develop and are often associated with off-flavor production [123,124,125]. Therefore, a careful management of the fermentation process during storage is increasingly being applied by distilleries, often employing marc acidification [122], temperature control [125], and yeast inoculation [126]. Research studies showed that the lowering of the pH [122] caused significant changes in the yeast–bacteria populations ratio and in yeast species turnover, determining an improvement of the aromatic profile of the distillate, due to the reduction of the main volatile products associated with potential off-flavors [122]. A significant impact on yeast ecology variability under marc storage and on sensory quality of the distillate was also shown for temperature [125]. Moreover, results demonstrated that effective inoculation of yeast strains (although not easy to achieve due to the solid state of grape marc) has a great impact on the fermentation of grape marc during storage, by leading not only to the increased development of aroma molecules, but also to the control of spoilage microorganisms that could greatly affect product quality [126].

Grape and wine co-products are also good sources of carbon and have been used to generate various high-value products like citric acid, lactic acid, gluconic acid, and ethanol through submerged and solid-state fermentation [127,128]. *Trichoderma harzianum, Aspergillus niger, Penicillium chrysogenum*, and *Penicillium citrinum* have been used in order to degrade winery biomass, leading to the production of commercially important metabolites such as, among others, stigmasterol, glycerol, maleic acid, xylitol, and citric acid [129]. Moreover, protein-rich products can be used as feedstock for animals. The protein content of grape marc increased from 7% to 27% in five days using the solid-state fermentation process and certain fungal strains and managing specific conditions like temperature and moisture content [130,131]. Submerged fermentation of grape wastes using *Monascus purpureus* produced a red pigment to be used in food industry and the usage of *Lactococcus lactis* and *Lactobacillus pentosus* led to the production of lactic acid and *Trametes pubescens* to produce laccase [131].

Finally, there is interest in wine co-products as a substrate for *Aureobasidium pullulans* growth and the production of pullulan, an extracellular and unbranched homopolysaccharide useful for biofilms and for applications in medical sciences, particularly drug delivery [51]. Grape skin pulp is considered as one of the best substrates for pullulan production, especially hot water extracts of the pulp. The product is of higher molecular weight and rather pure. Moreover, poly(β-L-malic acid) (PMA) is a natural biopolyester produced by many microorganisms including *A. pullulans*. The interest in this molecule could be attributed to its properties of being biodegradable, water-soluble, and biocompatible, and its uses in the pharmaceutical industry. No applications in the wine industry have been reported, but possible relationships can be explored via wine wastes as substrates for PMA production and PMA as a coating for post-harvest protection of grapes, similar to that previously proposed for pullulan production [51].

As a last remark, grape pomace (pulp and skins) was also investigated as a new biosorbent for removing mycotoxins from liquid media. In vitro adsorption experiments showed that the pomace obtained from Primitivo grapes was able to rapidly and simultaneously sequester different mycotoxins. Aflatoxin B_1_ was the most adsorbed mycotoxin, followed by zearalenone and ochratoxin A [132]. An innovative winemaking procedure involving the use of grape pomace has been suggested as a corrective measure to reduce ochratoxin A (OTA) levels in must and wines.

### 7.2. Microbial Treatment of Wastewater and Solid Residues

Wastewater sources are major causes for environmental pollution in surface and ground water bodies. Current wastewater treatment technologies are not sustainable because they are energy- and cost-intensive, leaving latitude for the development of technologies that are energy-conservative or energy-yielding. For the present and future context, microbial fuel cell technology may present a sustainable and environmentally friendly route to meet the water sanitation needs [133]. Microbial fuel cells (MFCs) are electrochemical devices that use the metabolic activity of microorganisms to oxidize fuels, generating electric current by direct or mediated electron transfer to electrodes. In the anodic compartment, organic matter is oxidized by microbial metabolism, which transfers the electrons to the anode. In the cathodic compartment, oxygen or oxidized compounds are reduced either via an abiotic process or by microbially mediated reduction [134]. The bacterial communities that develop in these systems show great diversity, ranging from primarily δ-Proteobacteria that predominate in sediment MFCs to communities composed of α-, β-, γ-, or δ-Proteobacteria, Firmicutes, and uncharacterized clones in other types of MFCs [135]. Microbial fuel cells can treat agro-industrial wastewater, and a few studies have reported the treatment of winery wastes. Dual chamber MFCs were used to treat real effluents from wine-processing factories. Results demonstrated that electricity can be produced efficiently and that the unbalanced nutrients/COD ratio was a major challenge in the treatment of winery wastewater, in spite of the very high organic load contained in this type of wastewater [136,137]. In another study, a single-chamber MFC was used to treat white wine lees and red wine lees [138]. Different degradability, due to different substrate composition, gave different results: white wine lees produced much more electricity and degradation, i.e., total-COD removal, than red wine lees (the high presence of polyphenols in the latter, played a role in reducing MFC performance). Different substrates led, also, to different microbial consortia. Electricity and degradation obtained with white wine lees indicated their suitability to be treated by MFC. At present, the technology is proposed for wineries at the industrial level for feasibility studies [139].

Besides, wine production determines the creation of large amounts of solid residues, such as vine branches from winter pruning and grape marc from winemaking (when not transferred to distilleries). Composting is a process transforming the organic matter by an aerobic biological process, allowing organic matter degradation and stabilization. Composting is becoming an ecological and economical microbially based alternative for reusing plant biomass residues [140] and therefore, residues from vitiviniculture. Indeed, by-products such as pruning residues and grape marcs can be exploited for the production of compost. In particular, grape marc [141,142] and branches have been composted, separately or together [143], showing beneficial results. Quite recently, wine by-products utilization by co-composting with olive mill wastewater was also proposed with promising results [144]. This represents an affordable and useful tool for both grape growers and winemakers, as the reutilization of residues produced by winemaking-related activities can lead to obtain a compost that can be reintroduced in the vineyard from where the plant biomass came from [143,145]. Additionally, it is worth to note that suppression of soil-borne diseases of horticultural crops by compost has been proved and attributed to the activities of antagonistic microorganisms, as a great diversity of biological control agents naturally colonize compost [140]. Focusing on winemaking by-products, the suppressive capacity of grape marc compost against *Pythium aphanidermatum* and *Phytophthora parasitica* was determined with promising results on cucumber [146]. In a further study, the large number of microbes which appeared in the microbiological analyses of grape marc compost was characterized and most microorganisms were bacteria. Antagonist in vitro assays were performed showing effective antagonistic activities against all the fungal pathogens tested [141]. This opens up further positive implications in terms of sustainability improvement. Most soil-borne pathogens are difficult to control by conventional strategies such as the use of synthetic fungicides. The lack of reliable chemical controls, the occurrence of fungicide resistance in pathogens, and the breakdown or circumvention of host resistance by pathogen populations are among the key factors underlying efforts to develop more sustainable control measures [141]. Therefore, the use of winery compost would represent an environmentally friendly tool, even in terms of circular economy if applied on vineyards [145].

## 8. Conclusions

In agriculture, the wine sector is one of the industries most affected by the environmental sustainability issue. Recently, the contribution of winemaking, from grape harvest to bottling, has been considered together with vineyard management in assessing the environmental impact of vitiviniculture. Several cellar processes could be improved for reducing the environmental impact of the whole chain, including microbe-driven transformations. The aim of this paper was to review the potential of microorganisms and interactions thereof as a natural, environmentally friendly tool to improve the sustainability aspects of winemaking, all along the production chain, including waste treatment. Microorganisms play a role in several steps of the winemaking process, most of which can be improved for reducing the environmental impact. These steps include pre-fermentative stages, alcoholic and malolactic fermentations and their management and timing, post-fermentation and stabilization processes, and valorization or treatment of by-products. Microbial resources exploitable for sustainability improvements include a wide array of genera and species comprising yeasts (both non-*Saccharomyces* and *Saccharomyces*), fungi, and bacteria. Moreover, microbial interactions and their exploitation also play a crucial role. Special attention was paid to microbial resources and processes which are already, or about to become, available for the winemaking sector at the industrial scale. In conclusion, the paper illustrates how the presence of proper yeast or bacterial strains, the management and timing of starter cultures inoculation, and some appropriate technological modifications that favor selected microbial activities can lead to several positive effects, including (among others) energy savings, reduction of chemical additives such as sulfites, and reuse of certain residues.

## List of abbreviations

ADYActive dry yeastAFAlcoholic fermentationBABiogenic amineCODChemical oxygen demandGHGGreenhouse gasLABLactic acid bacteriaMLFMalolactic fermentationMFCMicrobial fuel cellsOIVInternational Organisation of Vine and WinePCMPre-fermentative cold macerationVOCVolatile organic compound
